# The effect of transactional analysis training on emotional intelligence in health professions students

**DOI:** 10.1186/s12909-022-03455-y

**Published:** 2022-05-19

**Authors:** Hui Yean Seow, Mabel Huey Lu Wu, Mandakini Mohan, Norul Hidayah binti Mamat, Hildegunn Ellinor Kutzsche, Allan Pau

**Affiliations:** 1 Present Address: Private Dental Practitioner, Kuala Lumpur, Malaysia; 2 School of Dentistry, International Medical University, Bukit Jalil, Kuala Lumpur, Malaysia; 3 IMU Centre for Education, International Medical University, Kuala Lumpur, Malaysia; 4 IMU Clinical Skills and Simulation Centre, International Medical University, Kuala Lumpur, Malaysia; 5 Norwegian Women’s Public Health Association, Oslo, Norway

**Keywords:** Emotional intelligence, Health professions, Quasi-Experimental Study, Transactional analysis

## Abstract

**Background:**

Emotional intelligence (EI) is considered to present a significant predictor of work performance whereas Transactional analysis (TA) is the relational perspective in communication in managing emotions. We evaluated the effect of psycho-educational training in EI and TA (TEITA) on EI among health professions undergraduates, with post-training, and at 1-month follow-up.

**Methods:**

A total of 34 participants participated in the study where 17 participants were in the TEITA group and another 17 were in the control group. A quasi-experimental non-randomised, controlled cohort study was conducted, in which participants in the TEITA group were introduced to EI and TA concepts on a weekly basis for four weeks, at 90 min each time, and provided with opportunities for experiential sharing of emotions and coping mechanisms experienced in the previous week. Both TEITA and control groups received weekly EI and TA reading materials. All completed the 16-item Wong and Law EI Scale at baseline and post-training. The training group also completed the questionnaire at a 1-month follow-up. Wilcoxon Signed Ranks and Mann Whitney tests were used to analyse within a group and between group changes in EI scores.

**Results:**

Baseline EI scores in the TEITA group were lower than the control group. On completion of TEITA, EI scores in the TEITA group increased, and differences were not detected between groups. Within the TEITA group, paired increases in all domains were statistically significant, whereas, in the control group, the paired increase was only detected in the domain addressing regulations of emotion (ROE). Pre to post-training increases in EI scores were statically significantly greater in TEITA compared to control groups. At the 1-month follow-up, EI scores were sustained.

**Conclusion:**

The psycho-educational training based on EI and TA is effective in enhancing EI among health professions undergraduates. Future research should investigate the effect of such training on observable inter-personal and socio-economic behaviours.

## Background

Emotional intelligence (EI) has been termed as a set of abilities for sophisticated information processing about emotions and emotion-relevant stimuli, and using this information as a guide to thinking and behavior [1]. It has been related to educationally and socially relevant outcomes and theoretically linked to academic performance [2, 3], workplace effectiveness, including doctor-patient relationship and leadership [4–6], and psycho-social well-being [7, 8]. It has been suggested that personal growth and development, through emotional learning and maturation in emotional intelligence, are central to developing professional competence. Furthermore, EI has been recommended to be a measure of performance and an indicator of burnout besides being a selection criterion for training roles in healthcare [9, 10]. Recent literature provides an understanding of its importance in healthcare including patient satisfaction as well as possible economic implications [11–14]. This led to more research needed to determine whether EI can be improved through training and augmenting educational and clinical outcomes [4]. More recently, evidence has emerged to support the proposition that EI can be enhanced through psycho-educational training. The training in turn has the ability to lead to other favorable effects[15, 16]. Henceforth, the need to develop such training programmes to promote EI abilities, such as communication and conflict resolution has been called for [7] and the current global pandemic has made its relevance paramount [17].

The integration of EI training into medical and health professions education aims to contribute to developing professional [18, 19] and communication skills [20]. Interventions involving simulated patients [21], mental health awareness training [22] and leadership training [23] are effective to promote EI. An interesting consideration in the development of training to enhance EI in communication and interpersonal skills is the incorporation of transactional analysis (TA) training, as EI goes hand in hand with TA and has been considered a key training tool for communication skills*.* EI and TA have been appreciated as a learning method for training healthcare workers in communication and leadership skills [24, 25]. In a recent study, group therapy among drug addicts with the TA approach showed significant effects on EI expressed by coping capabilities [26].

The socio-psychological theory of TA explains that ego states are parts of our personality, systems of thinking, behaviors, and feelings and can be identified as the ego states of the Parent, the Adult and the Child. None of the above ego states ranks above the other, but we need to align the ego states with the situation we are faced with. The Parent ego state reflects parental thoughts and feelings; the Adult ego state relates to thoughts and feelings based on our own experiences, information, personal process and previous experiences; and the Child ego state is based on thoughts and feelings replayed from childhood [27]. TA provides a systematic approach that helps understand the link between human needs and behaviors, and the way that individuals, groups and organizations are effective or ineffective in communicating. It has been used to explain the psycho-emotional exploitation that can take place during patient encounters and result in the emotional exhaustion of clinicians [28]. Similarly, its relevance in understanding the challenges that can emerge in the clinical supervision context [29] as well as offering insight into our behaviours and relationships [27] has been reported. In clinical supervision, TA can be used to enhance the understanding of interpersonal interactions and improve the outcomes of clinical training [30]. Training in TA tools has been shown to result in sustainable and measurable improvement in self-awareness [31], a domain that is recognized in the EI framework. The basis for TA and the ego-state types in developing communication skills training for medical [32] nursing [33] and pharmacy students [34] has been recommended.

The literature would appear to suggest that both the TA tool and the EI ability have useful applications in interpersonal professional and social encounters. In such encounters, in which a transaction takes place between two or more people, the TA tool may be used to identify the ego states at play and the EI ability used to assess the emotions that are expressed or suppressed. The application of TA and EI may therefore help to enhance constructive communication and teamwork.

Undergraduate students may not have yet developed their ability to deliver safe and compassionate health care, a particularly pertinent issue in health professions education. With this background in mind, we sought to study emotional intelligence as a measurement of undergraduates' ability to socialize or relate to others, meaning balancing educational influences in undergraduate training to enhance patient-focused, social relationships and interactions made after considering the underlying motivation of our students.

We hypothesised that the psycho-educational TA training course on EI can be applied for self-emotional appraisal, others’ emotional appraisal, uses of emotion, and regulation of emotion, thereby contributing to a holistic personal growth. In this study, we tested the effect of psycho-educational training in EI and TA (TEITA) on changes in EI scores between baseline and on completion of TEITA, and at the 1-month follow-up.

## Methods

A quasi-experimental [35] non-randomised controlled cohort study was conducted on health professions undergraduates at one university campus to compare the baseline, post-training, and 1-month follow-up of EI scores of participants who completed a four-week psycho-educational training in EI and TA (TEITA) and those given reading materials on TA and EI. Institutional ethics approval was obtained (BDS I1-13(13)-2016).

The TEITA involved four weekly 90-min sessions designed by members of the research team (PKH, KHE, MNH). The TEITA aimed to introduce participants to EI and TA and support experiential sharing of emotions experienced in the previous week and mechanisms used to cope with those emotions. Each session included a discussion on various aspects of the EI and TA concepts for 30-45 min, along with 45 min to 1 h of experiential sharing. Each session was facilitated by two psychologists (KHE, MNH) and two research assistants (SHY, WHL) (Table 1). Both the TEITA and control groups received reading materials on TA and EI on a weekly basis. The reading material included articles and internet resources.

**Table 1:** Schedule of TEITA Delivery

Session 1	6.00 – 6.15 pm	Explanation of ground rules	SHY and WHL
	6.15 – 6.30 pm	PowerPoint presentation on EI	SHY
	6.30 – 6.40 pm	Ice breaking game	KHE and MNH
	6.40 – 7.30 pm	Experiential sharing	KHE and MNH
	7.30 pm	Wrap up and summary	SHY and WHL
Session 2	6.00 – 6.25 pm	Review on previous session	SHY and WHL
	6.25 – 6.40 pm	PowerPoint presentation on TA	WHL
	6.40 – 7.30 pm	Experiential sharing	KHE and MNH
	7.30 pm	Wrap up and summary	SHY and WHL
Session 3	6.00 – 6.40 pm	Ego states determination by using TA Questionnaire	KHE and MNH
	6.40 – 7.30 pm	Experiential sharing	KHE and MNH
	7.30 pm	Wrap up and summary	SHY and WHL
Session 4	6.00 – 7.00 pm	Experiential sharing	KHE and MNH
	7.00 – 7.15 pm	Post intervention questionnaire	SHY and WHL
	7.15 – 7.30 pm	Wrap up and summary	SHY and WHL

The sampling population included health professions medicine, dentistry, pharmacy and psychology undergraduates at a university. Based on a 95% confidence level and 80% power, a sample size of 34 subjects (17 in control and 17 in TEITA groups) was estimated to detect a difference of 50% in the proportion of subjects increasing their EI scores between baseline and post-training. This assumed that 10% of the control participants and 60% of the TEITA participants would have increased their EI scores from baseline to post-training.

Potential participants were approached by email to voluntarily participate in this study. Flyers were placed around the university campus and on the screensaver on university computers. Information given to potential participants included the aim of the research, the time and venue, the number of participants required, the benefits of participating in the research, as well as the contact researcher’s information. Participants who volunteered and consented in writing to receive TEITA were assigned to the training group, whereas those not interested in TEITA were assigned to the control group. Participants assigned to the training group were divided into 2 small groups, each receiving the TEITA separately. Informed consent for the confirmation of autonomy among all participants in this study was signed and all relevant research information was provided. The participant was given the opportunity to withdraw from the research at any time and for any (or no) reason.

The EI instrument used was the 16-item self-report Wong and Law EI Scale (WLEIS) measured on a 7-point Likert-type scale [36], from 1 = totally disagree to 7-totally agree. These 16 items are distributed into four domains: self-emotional appraisal (SEA), others’ emotional appraisal (OEA), use of emotion (UOE), and regulation of emotion (ROE). The WLEIS has since been widely validated across different contexts and in different languages [37–40]. All participants completed the WLEIS at baseline and on completion of the 4-week TEITA. The training group also completed the WLEIS at the 6-month follow-up.

### Data analysis

The Statistical Package for Social Sciences (SPSS) software for Windows, Version 24.0 was used for statistical analysis. Sample characteristics between the training and control groups were compared using the Chi-square test for distribution by sex, and the Mann–Whitney test for differences in age and EI scores. Baseline and post-training EI scores between training and control groups were compared using the Mann–Whitney test. Paired changes in EI scores from baseline to completion of TEITA within the training and control groups were tested using the Wilcoxon Signed Ranks test. The primary outcome of changes in EI scores from baseline to post-training was compared between the training and control groups. Paired changes in EI scores from baseline to 1-month follow-up for the training group were tested for statistical significance using the Wilcoxon Signed Ranks test. Statistical significance was set at *p* = 0.05.

## Results

The present study introduced a sample of undergraduates to the concepts of EI and TA on a weekly basis for four weeks, at 90 min each time, and provided opportunities to apply the concepts of TA and EI to social encounters experienced in the previous week. Students also explored the emotions that they felt during these encounters and discussed mechanisms used to cope with those emotions.

Of the 19 (55.9%) females enrolled, 14 (73.7%) volunteered for the TEITA compared to 3 (20.0%) of the 15 (44.1%) males (*p* = 0.002). The median age (inter-quartile range) of participants in the intervention group was 22 (19-23) and 22 (21-23) in the control group (*p* = 0.156) (Table 2).

**Table 2:** Baseline comparison between intervention and control groups by sex, age and EI scores

	Intervention (*n* = 17)	Control (*n* = 17)	*p*-value
Female (*n* = 19, 55.9%)	14 (73.7%)	5 (26.3%)	0.002
Male (*n* = 15, 44.1%)	3 (20.0%)	12 (80.0%)	
Age in years: median (IQR)	22 (19–23)	22 (21–23)	0.156
EI Total: median (IQR)	75.0 (62.0–81.0)	82.0 (77.0–90.0)	0.027
SEA	19.0 (17.5–21.5)	23.0 (20.5–24.0)	0.005
OEA	18.0 (13.0–20.0)	20.0 (17.5–23.5)	0.049
UOE	20.0 (15.5–22.0)	20.0 (17.5–23.0)	0.578
ROE	17.0 (15.0–21.0)	19.0 (18.0–22.0)	0.107

At baseline, males scored more highly in SEA (0.045) compared to females, but both groups were similar for EI Total (0.266), OEA (0.689), UOE (0.990), and ROE (0.062) (Table 3). On completion of TEITA, no difference between the two groups was detected. Among the females, statistically significant paired increases in EI Total and all domain scores were detected. Similarly, paired increases were detected among males. However, increases in EI and all domains’ scores from baseline to completion of TEITA were not statistically significantly different between the two groups.

**Table 3:** Comparison of EI scores between and within females and males

	Pre		Pre-scores between groups	Post		Post-scores between groups	Paired score changes within females	Paired score changes within males	Post–Pre		Post–Pre score changes between groups
	Female	Male	^a^*p*-value	Female	Male	^a^*p*-value	^b^*p*-value	^b^*p*-value	Female	Male	^a^*p*-value
EI Total	77.0 (71.0–83.0)	81.0 (73.0–92.0)	0.266	86.0 (81.0–83.0)	85.0 (78.0–97.0)	0.781	0.001	0.002	9.0 (4.0–19.0)	7.0 (1.0–11.0)	0.348
SEA	20.0 (18.0–21.0)	24.0 (19.0–24.0)	0.045	23.0 (22.0–25.0)	24.0 (21.0–25.0)	0.986	0.006	0.048	3.0 (0.0–7.0)	1.0 (0.0–6.6)	0.136
OEA	19.0 (15.0–23.0)	18.0 (16.0–21.0)	0.689	23.0 (19.0–24.0)	20.0 (18.0–24.0)	0.637	0.021	0.008	1.0 (0.0–6.0)	2.0 (0.0–5.0)	1.000
UOE	20.0 (19.0–23.0)	20.0 (16.0–23.0)	0.930	22.0 (20.0–24.0)	21.0 (19.0–23.0)	0.329	0.005	0.024	3.0 (0.0–5.0)	1.0 (0.0–2.0)	0.186
ROE	18.0 (15.0–20.0)	22.0 (18.0–24.0)	0.062	21.0 (18.0–23.0)	24.0 (19.0–23.0)	0.215	0.005	0.015	2.0 (-1.0–5.0)	2.0 (0.0–3.0)	0.625

^a^Mann-Whitney Test for differences between groups

^b^Wilcoxon Signed Ranks Test for paired differences within group

At baseline, the control group scored more highly in EI Total (0.027), SEA (0.005) and OEA (0.049) (Table 4). Both groups scored similarly in UOE (0.578) and ROE (0.107). However, on completion of TEITA, both groups scored similarly in EI Total and all its domains. Within the training group, paired increases in EI Total and all domain scores were statistically significant. Within the control group, paired increases in EI Total (0.012) and ROE (0.010) were statistically significant. Increases in scores from baseline to completion of TEITA were statically significantly different between the two groups for EI Total (0.002), SEA (0.001), OEA (0.027) and UOE (0.017), but not ROE (0.252).

**Table 4:** Comparison of EI scores between and within intervention and control groups

	Pre		Pre-scores between groups	Post		Post-scores between groups	Paired score changes within intervention	Paired score changes within control	Post–Pre		Post–Pre score changes between groups
	Intervention	Control	^a^*p*-value	Intervention	Control	^a^*p*-value	^b^*p*-value	^b^*p*-value	Intervention	Control	^a^*p*-value
EI Total	75.0 (62.0–81.5)	82.0 (77.0–90.0)	0.027	86.0 (80.0–99.0)	85.0 (80.0–92.5)	0.605	0.001	0.012	13.0 (8.0–26.5)	4.0 (0.0–8.0)	0.002
SEA	19.0 (17.5–21.5)	23.0 (20.5–24.0)	0.005	24.0 (22.0–26.0)	23.0 (21.0–24.5)	0.259	0.002	0.586	5.0 (1.0–7.0)	0.0 (0.0–1.0)	0.001
OEA	18.0 (13.0–20.0)	20.0 (17.5–23.5)	0.049	23.0 (19.0–24.0)	21.0 (18.5–24.0)	0.702	0.004	0.057	6.0 (0.5–8.0)	0.0 (0.0–2.5)	0.027
UOE	20.0 (15.5–22.0)	20.0 (17.5–23.0)	0.578	22.0 (20.0–25.0)	21.0 (19.0–22.5)	0.219	0.002	0.116	3.0 (1.0–5.0)	0.0 (0.0–2.5)	0.017
ROE	17.0 (15.0–21.0)	19.0 (18.0–22.0)	0.107	21.0 (18.0–23.5)	21.0 (19.5–24.0)	0.466	0.004	0.010	3.0 (0.0–8.0)	2.0 (0.0–3.0)	0.252

^a^Mann-Whitney Test for differences between groups

^b^Wilcoxon Signed Ranks Test for paired differences within group

At the 1-month follow-up, EI Total and domains scores in the training group remained statistically significantly higher than at baseline (Table 5), confirming that increases in EI scores were sustained a month later.

**Table 5:** Intervention group EI scores at 1-month follow-up (FU) and changes from baseline

	1-month FU	1-month FU—Baseline	^b^*p*-value
EI Total	89.0 (77.0–95.5)	16.0 (10.5–25.5)	0.007
SEA	23.0 (21.0–24.5)	5.0 (1.0–6.5)	0.011
OEA	21.0 (19.5–24.5)	4.0 (1.0–9.0)	0.010
UOE	23.0 (18.5–26.0)	3.0 (1.5–7.0)	0.030
ROE	20.0 (17.0–24.5)	4.0 (-0.5–7.0)	0.046

^b^Wilcoxon Signed Ranks Test for paired differences within group

## Discussion

This study sought to investigate whether the cultivation of EI and TA educational training in a group of health professions students can afford students the opportunity to realise improved EI and TA capacities immediately after training and at the 1-month follow-up. The findings suggest that exposure to the four weekly 90-min training sessions resulted in an increase in EI outcomes immediately after educational training with a persisting effect one month later. The concept of EI has generated interest in terms of its relevance to well-being regulation, academic performance and occupational effectiveness. EI and TA have often formed the theoretical basis for understanding inter-personal dynamics in communication [33, 41, 42] and leadership [6, 27, 43, 44] skills. Training based on both theoretical underpinnings has been reported to result in positive outcomes [21, 31]. In the current study, the experiential sharing part of the TEITA offered the opportunity to understand the structure and functioning of the participant’s personality and recognize how they respond to others. By listening and watching how others share their experiences, group members learned to identify their own emotions better. This training programme took advantage of the synergistic learning experience of both concepts [24] and the result was a sustained increase in EI among undergraduates who took part in the training. Previous research has similarly reported the effectiveness of TA training in enhancing EI and empathy. In one study exposing 16 nurses to a psycho-educational training on TA, the results showed a positive impact on participants’ communication skills and empathy scores [24]. In another study, 15 subjects participated in 12 sessions of TA-based group psychotherapy and were shown to have increased EI compared to subjects who were not exposed to the intervention [26]. A study conducted on 19 medical teachers who underwent a 12-h TA training, reported an improved self-awareness that persisted at three months and one year after training. The study concluded that improved personal ‘awareness’ among teachers can improve the educational environment and is essential to foster student learning [31].

Participants in Kanchana et al.’s study showed a significant increase in post-intervention scores on all four dimensions of emotional intelligence using a 2-day internationally recognized TA course on 101 participants [45]. Results from the present study suggest that increases in EI scores in the training group were higher than in the control group in the SEA, OEA and UOE domains. In identifying the ego states manifested in an interpersonal transaction and any possible conflicts that may emerge, an individual can begin to appraise the emotions that are aroused in themselves and others. That individual can then decide on the next course of action based on the emotions they feel. However, no difference in improvement in the ROE domain was detected between the training and control groups. It is possible that development of the ROE skill may require a longer period of training and practice than that afforded by the present training programme. Further research is needed to explore training that can promote emotional regulation.

In the present study, more female students volunteered for the TEITA than male students. At baseline male subjects scored more highly in EI than female subjects. However, on completion of TEITA, EI scores in the females increased substantially such that differences with males lost statistical significance, and there were no differences in EI scores changes between females and males. Since females were over-represented in the TEITA group, increases in EI scores were likely to be due to the training. This highlights a limitation of the present study; it was not able to show whether training was able to affect EI improvement in male subjects. Although it has been reported that females are more likely to benefit from EI training [21], the influence of gender on EI training shows inconclusive results in literature [46].

A non-randomized study approach was chosen for our post-intervention study for the reason of the difficulty of randomizing subjects and small available sample size. The risk of self-selection bias in non-randomised studies can lead to biased estimates in terms of a balance of motivation in investing and completing the training [47]. However, this lack of balance had only a small impact on the outcome because, the EI scores of the TEITA subjects at baseline were lower than the control subjects, and significant increases in EI scores were clearly detected post-training. Furthermore, improvement in EI scores was sustained at the 1-month follow-up. This is a strong point of the study as follow-up measurement is purported to be an important criterion for evaluating EI training [16]. While the small sample size remains a limitation of the study, the participants included a mix of undergraduates from different health professions courses at the university. Though many studies have reported on the effects of TA in nursing, there is a paucity of current literature on its role in other health professions. This study reports on an opportunity for interprofessional learning that may promote the development of interdisciplinary teamwork [48] which is so critical in healthcare.

## Conclusion

In conclusion, within some limitations, psycho-educational training based on EI and TA has been shown to be effective in enhancing EI in health professions students. Future research is needed to test this intervention in different populations. Research should also investigate the effect of such TA and EI training on observable inter-personal and socio-economic behaviours as well as the sustained retention of the effect of such training.

## Data Availability

All data generated or analyzed during this study are included in this published article.

## Abbreviations

EI: Emotional Intelligence
TA: Transactional analysis
TEITA: Training in emotional intelligence and transactional analysis
WLEIS: Wong and Law Emotional Intelligence Scale
SEA: Self-emotional appraisal
OEA: Others’ emotional appraisal
UOE: Use of emotion
ROE: Regulation of emotion
